# The Canine Vaginal Flora: A Large-Cohort Retrospective Study

**DOI:** 10.3390/vetsci11020055

**Published:** 2024-01-27

**Authors:** Anna Sophia Leps, Babette Klein, Marianne Schneider, Cornelia Meyer, Alexandra Šoba, Christine Simon, Viktor Dyachenko, Ute Siesenop, Jutta Verspohl, Sandra Goericke-Pesch

**Affiliations:** 1 Unit for Reproductive Medicine—Clinic for Small Animals, University for Veterinary Medicine Hannover, Foundation, Bünteweg 15, 30559 Hannover, Germany; anna.sophia.leps@tiho-hannover.de; 2 Laboklin GmbH & Co. KG, 97688 Bad Kissingen, Germany; 3 SYNLAB Vet, an Antech Company, 86156 Augsburg, Germany; 4 Biocontrol, Biosciencia Healthcare GmbH, 55218 Ingelheim, Germany; 5 Institute for Microbiology, University of Veterinary Medicine Hannover, 30173 Hannover, Germany

**Keywords:** vaginal flora, bitch, breeding management

## Abstract

**Simple Summary:**

To obtain a better understanding of the canine vaginal flora, we analyzed the results of 23,254 vaginal swab samples that had been sent for conventional aerobic bacterial culture to three commercial laboratories between 2015 and 2021. The results show that the canine vagina is inhabited by numerous microorganisms, mostly in mixed cultures of two or more bacterial isolates. Our study confirms the variability of the canine aerobic bacterial flora described in earlier studies, however, for the first time in a vast population.

**Abstract:**

Microbiological examinations are frequently performed as part of breeding management examinations in the bitch, but also in case of (suspected) reproductive tract problems. As most bacteria are opportunistic pathogens, evaluation of bacterial findings is challenging for veterinarians. Besides, breeders might request antimicrobial treatment in breeding bitches, fearing conception failure—even without medical indication. Considering the rising threat of antimicrobial resistance, gaining deeper insights into the bacterial findings from the vagina of healthy and (suspected) reproductive-diseased bitches might contribute to the knowledge of the canine aerobic vaginal flora and consequently improve the responsible use of antibiotics. We analyzed results from bacteriological cultures of 23,254 vaginal swabs sent in to three commercial laboratories in Germany between 2015 and 2021, where standard aerobic microbiological examination was carried out. We found a variety of 319 bacterial species that mostly grew in mixed cultures of two or more bacterial species. Commonly found species were *Escherichia coli*, beta-hemolytic Streptococci, coagulase-positive Staphylococci, Pasteurellales, and aerobic sporulators, as well as other *Streptococcus* spp. Our results showed a large diversity of the canine vaginal flora in healthy and (suspected) reproductive-diseased bitches. They largely support earlier findings of small studies on the physiological canine vaginal flora, emphasizing that solely the results of a bacterial evaluation should not be the base for antimicrobial treatment. Instead, bacterial findings should be evaluated with the results of a clinical gynecological examination.

## 1. Introduction

Microbiological culture of the vaginal flora prior to mating is frequently performed during breeding management in the bitch. Among many breeders, the conception that the presence of bacteria in the vagina of the bitch is invariably associated with infection and possible infertility exists until today. Fearing unsuccessful mating because of bacterial inhabitation of the vaginal mucosa, some breeders demand a prescription of antibiotics, even prophylactically [1,2]. Besides, despite several studies describing the physiological vaginal flora from bitches [3,4,5,6,7,8,9,10,11,12,13], many veterinarians are not confident about how to interpret findings of bacterial culture of the vagina—which bacteria to consider physiological and which (potentially) pathological. This insecurity and/or uncertainty of veterinarians might be due to different reasons: 1. Limited personal expertise in the field of small animal breeding management; 2. Limited knowledge of the physiological vaginal flora as the former studies included only a few animals, ranging from 9 to 143 animals [9,11]; 3. Limited ability to differentiate between physiological and pathological flora based on bacterial findings as most bacteria found in the female genital tract act as opportunistic pathogens [3,4]. As a consequence, antimicrobial treatment is often initiated just “to be safe” and not to be blamed in case of conception failure. However, it was repeatedly proven that the presence of bacteria without any clinical signs is not to be interpreted as proof of a reproductive tract infection or cause of infertility [3,4,5]. Although some species (e.g., *Escherichia coli*) could be associated with reproductive problems like pyometra and puppy death [5,14], other studies found that the sole presence of these bacteria, even in pure culture, did not necessarily coincide with pathologic findings in the reproductive tracts, neither clinically nor histologically [3,4,5]. Even further, the same species were also found in the physiological flora of healthy bitches. Some bacterial species might even play a “protective” role, as Groppetti et al. showed that the presence of *Streptococcus* spp. (except for beta-hemolytic Streptococci) during proestrus was negatively correlated with uterine infections during the luteal phase [3]. Non-indicated antibiotic treatment not only has the potential to worsen resistance development in general [15] and in the kennel, specifically [1], but it also bears the risk of worsening the clinical situation of the individual bitch when resistant bacteria proliferate in pure culture after application of antibiotics. Whereas it might be postulated that in mixed cultures, several strains of bacteria compete with each other for nutrients, etc., thereby preventing overgrowth and clinical signs, pure cultures are confirmed to be more often isolated from sites of infection [16]. Clinical consequences of infection caused by opportunistic pathogenic bacteria might be conception failure, embryonic resorption, and abortion [14,17,18]. Changes in the uterine milieu in the dog during the implantation period are subject to current research [19,20]. A shift of the inflammatory processes from pro-inflammatory in the pre-implantation period to rather anti-inflammatory conditions during implantation and trophoblast invasion suggests a role of immune cells in the establishment of pregnancy in the dog. Therefore, a changed uterine milieu—possibly associated with antibiotic treatment—could influence the establishment of a physiological pregnancy, not only in humans [21] but also in dogs [20]. Once antibiotics are administered, the sensitive balance of the microbial environment is disturbed, and the microbiome is significantly altered [1,22]. This is related to the fact that sensitive bacterial organisms are rapidly eradicated, whereas resistant bacteria remain [22]. The eradication of resistant and less-sensitive organisms might be unsuccessful. Moreover, the absence of competitors for nutrients might enable the proliferation of potential pathogens and the selection of resistant strains [1,2,3]. Nevertheless, the number of multidrug-resistant bacterial strains and nosocomial infections has risen over the past years [23], and antimicrobial stewardship has become more important than ever. A One Health approach is needed to face this challenge, and both human and veterinary medicine, including infectious disease and microbiology specialists as well as clinicians, are required to take action [24]. Optimization of antimicrobial treatment and in particular avoidance of unnecessary treatments is important, as the latter increases the selective pressure for antimicrobial resistance [15]. Still, decision-making regarding the use of antimicrobials sometimes is not easy. To facilitate the process, authorities and specialists in the field have published several guidelines on the use of antibiotics [25,26,27]. Considering the fact that bacteria in the canine vagina act opportunistically rather than obligate pathogenic (with the exception of *Brucella canis*) [18,28], diagnosis of reproductive disease only by microbiological examination results is impossible. This once more emphasizes the need for a proper clinical and gynecological examination along with the microbiology results to correctly interpret the findings. The aim of the study was to identify the common aerobic bacteria found on the canine vaginal mucosa to describe the canine vaginal flora in a vast population. Therefore, we analyzed a large cohort of 23,254 samples collected over a period of six years.

## 2. Materials and Methods

Retrospective analysis of microbiological examination results of 23,254 vaginal swabs was performed. Results were obtained from three commercial laboratories from sent-in samples collected by German veterinary practitioners between 2015 and 2021. The data provided by the laboratories included the date of collection, sample ID number, species, site of sampling (vaginal swabs), cultured isolates, grade of bacterial growth, and antimicrobial susceptibility test—if performed. Data for the grade of bacterial growth is lacking in single samples for unknown reasons, as shown in the results. Data were obtained and analyzed annually but also summarized over the whole time period. Data analysis was descriptive, and Microsoft Excel (Microsoft^®^ Excel, Version 16.74, Microsoft Corporation, Washington, DC, USA) was applied for data analysis and graphical presentation of data. After the samples were sent in, all laboratories carried out a standardized microbiological culture. The samples were streaked onto different non-selective and selective culture media. As non-selective media, Columbia Agar with 5% sheep blood (Becton Dickinson GmbH, Heidelberg, Germany/Oxoid GmbH, Wesel, Germany) and Chocolate Agar (Becton Dickinson GmbH) were used. Besides, Endo-Agar (Becton Dickinson GmbH), Columbia CNA-agar with 5% sheep blood, MacConkey-Agar (Becton Dickinson GmbH), and Biplate MacConkey-Agar/Columbia CNA-Agar with 5% sheep blood (Becton Dickinson GmbH were applied as selective media. Additionally, every sample was enriched in either 8 mL Thioglycolate-Bouillon or Tryptone-Soya-Bouillon (Becton Dickinson GmbH). The media were incubated at 35–37 °C (±1 °C) under aerobic conditions. After 18–24 h, the cultures were first checked for growth. The second check was carried out after 48 h. Bacterial growth was classified as low-grade (+), intermediate (++), or high-grade (+++). To evaluate the grade of growth, a three-phase-streaking pattern was used. Growth in the first phase was classified by the investigator from “+” to “++”, in the second phase from “++” to “+++” and as “+++” in the third phase. Growth that occurred only after enrichment was specified as such. The diagnosis was based on cultural and biochemical parameters, as well as mass spectrometry (matrix-assisted laser desorption/ionization-time of flight, MALDI-TOF MS, MALDI Biotyper, Bruker Daltonics GmbH, Bremen, Germany). The Reference Library is subject to continuous updates, and the latest versions were used to identify bacterial species. Morphology of the colonies was evaluated on selective agar plates and biochemical parameters were examined via catalase test (3% solution, LABOKLIN GmbH, Germany and SYNLAB Vet, an Antech Company), oxidase test (MAST-ID Oxidase-Teststreifen, MAST Diagnostica GmbH, Reinfeld, Germany) and latex agglutination (DiaMondial Staph Plus Kit, DiaMondial, Langenhagen, Germany and Pastorex Staph Plus, Bio-Rad Laboratories GmbH, Feldkirchen, Germany). For identification of an isolate colony morphology and biochemical parameters and/or MALDI-TOF MS was used. Identifications by MALDI-TOF MS are validated using a score. The spectrometric result of an examination is compared to a reference library of known species and scored for the probability [29]. A score of at least “2” was required for a result to be considered reliable.

Additionally, statistical analysis was carried out for the qualitative distribution of bacterial species according to groups over time, aiming to identify whether a significant difference exists for the overall occurrence of species (distribution) between the different years (2015–2021). Besides, we tested for selected bacteria/bacterial groups that were frequently identified and considered potentially clinically relevant, namely *Escherichia coli*, beta-hemolytic Streptococci, and coagulase-positive Staphylococci, whether significant differences exist over time regarding their growth intensity (only after enrichment/low-grade/intermediate/high-grade). For all statistical tests, Graph Pad Prism 9.0 (Graph Pad Software, Inc., La Jolla, CA, USA) was used. Initially, data was tested for normal distribution using the Shapiro-Wilk normality test. As all tested data was normally distributed, ANOVA was used to test for an overall difference over the years. Results were considered to be statistically significant at a level of *p* < 0.05. As ANOVA revealed no significant differences, no post-hoc tests were applied.

## 3. Results

In total, in 23,254 samples, 319 different bacterial species were found. Species were assigned to the following groups: coagulase-positive Staphylococci, coagulase-negative Staphylococci, gram-positive rods (other than *Bacillus* spp., *Lactobacillus* spp. and *Carnobacterium* spp.), beta-hemolytic Streptococci, *Enterococcus* spp., Enterobacterales (excluding *Escherichia coli*), *Escherichia coli*, gram-negative non-fermenters, Aeromonales, Pasteurellales (Table 1). The remaining 42 isolates do not all have a common phylogenetic background but are known as common commensals on canine mucous membranes. They were grouped as such (“common inhabitants of mucosae”, shown in Table 1), with rather clinical than phylogenetic reasoning.

### 3.1. Qualitative Analysis

**Figure 1:**
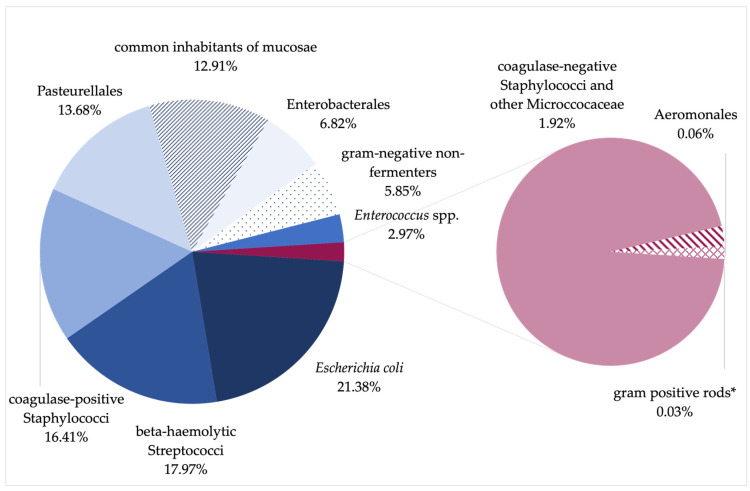
Percentage distribution of specified groups of bacteria isolated from canine vaginal swabs. * Gram-positive rods, other than *Bacillus* spp., *Lactobacillus* spp., and *Carnobacterium* spp.

From all samples (n = 23,254), 319 different species were isolated. In all samples with bacterial growth, the most common bacterial species was *Escherichia coli* (n = 7776; 21.4%), followed by beta-hemolytic Streptococci (n = 6535; 18.0%) and coagulase-positive Staphylococci (n = 5969; 16.4%). Pasteurellales were found in 13.7% of the samples (n = 4976). Common inhabitants of the mucosae made up a total percentage of 12.9% (n = 4695), of which 4% (n = 1450) could not be further differentiated as these samples contained “mucosal flora”, as defined by each laboratory. Other than that, Enterobacterales (n = 2482; 6.8%), gram-negative non-fermenter (n = 2129; 5.9%), and *Enterococcus* spp. (n = 1079; 3.0%) were found. Only a small group of samples showed growth of coagulase-negative Staphylococci and other Micrococcaceae (n= 697; 1.9%), Aeromonales (n = 21; 0.06%), and gram-positive rods (other than *Bacillus* spp., *Lactobacillus* spp., and *Carnobacterium* spp.) (n = 13; 0.04%) (Figure 1).

### 3.2. Number of Species Per Sample

The number of isolates per sample was defined in 21,125 samples. In the remaining samples (n = 2129), isolates were either summarized as “mucosal flora” (n = 1263) by the laboratories (the exact number of identified bacterial species in these samples is unknown) or showed no growth (n = 866) (Table 2). In the majority of samples (52.6%, n = 12,230), a mixed flora was identified with mostly two species (35.7%, n = 8302), but occasionally also more than four species (0.1%, n = 16) per sample. Monoculture was found in 43.7% of samples (n = 10,158).

### 3.3. Qualitative Analysis of Monocultures

Similar to the qualitative analysis of all samples, *Escherichia coli* (29.3%, n = 2976) was also the most common bacterial species among the monocultures (n = 10,158). Besides, Pasteurellales (17.5%, n = 1779) were relatively frequently found in monoculture. Coagulase-positive Staphylococci (16.7%, n = 1695), beta-hemolytic Streptococci (15.6%, n = 1583), gram-negative non-fermenter (5.4%, n = 552) and *Enterococcus* spp. (1.9%, n = 191) were distributed in similar amounts compared to their overall appearance (Table 3).

### 3.4. Semiquantitative Analysis

**Figure 2:**
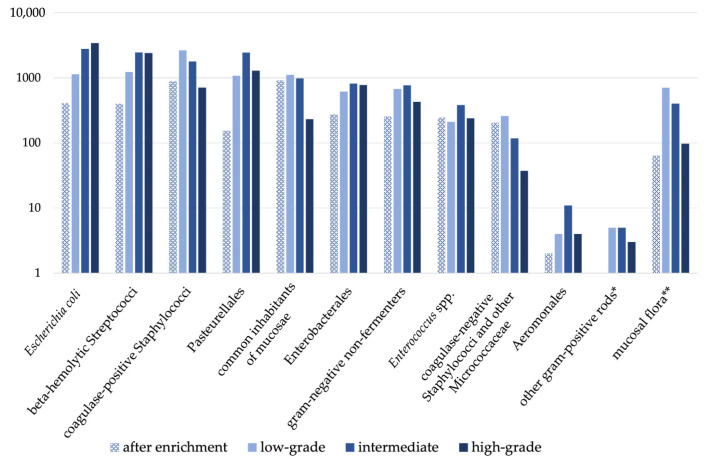
Semiquantitative scoring of grades of growth separately for the groups of species. Bacterial growth was graded as “after enrichment”, “low-grade”, “intermediate” and “high-grade”. * gram-positive rods, other than *Bacillus* spp., *Lactobacillus* spp., and *Carnobacterium* spp.,** mucosal flora, as defined by the laboratory.

Classification of bacterial growth was available for 36,098 isolates. It was not specified in 291 samples with bacterial growth for unknown reasons. While the majority of species in most groups showed low-grade to intermediate growth, a tendency for stronger growth (higher grades) became obvious for both *Escherichia coli* and beta-haemolytic Streptococci. Results are shown in Figure 2.

### 3.5. Comparative Annual Qualitative and Semiquantitative Analysis

Comparing the qualitative distribution of bacterial species based on the previously defined groups over time, no significant difference was identified (ANOVA, *p* = 0.629) supporting that distribution in all years (2015–2021) was similar (Table 4).

Comparing semiquantitative evaluation of bacterial growth for selected bacteria/bacterial groups that were frequently identified and considered potentially clinically relevant. Statistical data analysis revealed no significant difference over time. In detail, comparing annual results from 2015 to 2021 for *Escherichia coli*, beta-hemolytic Streptococci, and coagulase-positive Staphylococci ANOVA results were not significant (*Escherichia coli*: *p* = 0.2456, beta-hemolytic Streptococci: *p* = 0.6846, coagulase-positive Staphylococci: *p* = 0.6487).

## 4. Discussion

In this study, we retrospectively analyzed a large cohort of vaginal swabs to classify the canine vaginal bacterial flora. Our current results obtained from a large sample size support largely earlier results conducted on predominantly clinically healthy dogs and small study populations [3,4,5,7,8,10].

As expected and in good agreement with earlier studies [4,5,6,13], the majority of vaginal samples (96.3%) from our large cohort revealed bacterial growth. No bacterial growth was found in 3.7% of the samples. Comparing this to other studies, a maximum of 20.9% of samples of healthy dogs (9 out of 43 in total) did not reveal bacterial growth in the bacterial sample [3]. However, comparative vaginal cytologies were obtained in the respective study with bacteria identified in the examined slides, letting these authors suggest insufficient culturing of the microorganisms in the corresponding microbiological specimens [3]. Nevertheless, all these findings clearly support that the presence of bacteria in the canine vagina has to be considered physiological, independent of disease.

Despite recent culture-independent techniques such as next-generation sequencing became popular tools—also to investigate the vaginal flora [30] due to their capability to capture additionally hard-to-culture organisms of the microbial ecosystems [31,32,33,34,35], culture-based methods are still widely used (not only for data included in our study) and currently not replaceable. Important reasons for the ongoing need and use of culture-based methods in clinical microbiology are availability, costs of equipment and staff [32,33], and, most importantly, the possibility of antimicrobial susceptibility testing [35]. Different from sequencing methods that are able to detect genotypic resistance, only culture techniques are capable of determining the phenotypic resistance of the selected isolates of a species [34]. Today, the sole use of microscopy and biochemical parameters for the identification of bacterial species has been replaced in many laboratories [29], and MALDI-TOF-MS is routinely added for the identification of bacteria. As MALDI-TOF MS provides a highly accurate and rapid identification of bacterial species [36], even in many difficult-to-identify isolates with reference libraries being constantly updated, this method is considered one of the most important developments in microbiological diagnostics in the past decade [29], also in cases where conventional methods often lead to uncertain results [37]. The results included in our study are based on up-to-date aerobic clinical microbiology diagnostics, therefore considerably contributing to current knowledge. Compared to earlier studies using aerobic bacterial culturing [3,4,5,6,7,8,9,10,11,12,13], a larger variety of bacterial species was identified in our current study. This might be related to the larger number of samples assessed compared to earlier studies (the biggest study included 826 samples collected from 59 bitches over an 18-month course [4]) but also related to the methods used. Nevertheless, species and groups of bacteria identified most frequently in our present study (*Escherichia coli* and other Enterobacterales, Streptococci, Staphylococci, and Pasteurellales) accounted for the majority of bacteria in previous studies [4,9,13]. Interestingly and in good agreement with earlier analysis, a vast majority of isolates are common enteric organisms, suggesting a relation between inhabitants of the intestines and the genital tract—either as common inhabitants of the mucous membranes or due to licking of the anogenital region by bitches resulting in self-inoculation with the enteric bacteria. Without a doubt, based on our and previous data, it can be concluded that not only bacterial growth is physiological, but that also facultative pathogenic bacteria have to be considered as part of the canine vaginal flora. Noteworthy, no conclusions on the presence of *Mycoplasma* spp. are possible from the current dataset due to the used culturing techniques. As *Mycoplasma* spp. culturing is quite complicated and labor-intensive [38]; routine culturing and identification of these microorganisms is often not performed but rather replaced by PCR analysis (also in the laboratories providing data). Due to their (negative) “popularity” among breeders, their potential role in urogenital tract infections and infertility [13,39,40], but also due to the fact that *Mycoplasma* spp. are common inhabitants of the vaginal flora of the bitch [7,13,39,40], further research is of particular interest.

As expected, most samples (58.6%, n = 13,628) showed growth of multiple bacteria supporting that the canine vaginal flora is usually a mixed flora. On the other hand, 43.7% of samples revealed pure culture (monoculture). The clinical relevance of pure culture is still controversial, as the incidence of pure cultures in healthy animals varies considerably between studies, ranging from 27 to 62.5% [6,12]. Besides, there is some evidence that the occurrence of pure cultures might differ between cycle stages [12,13]. The majority of pure cultures in our study consists of *Escherichia coli* (29.3%; n = 2976), followed by Pasteurellales (17.5%; n = 1779), accounting for 38.3% and 35.8% of total isolates within the respective group of bacterial species. This partly corresponds to Bjurström et al. [4], as they found *Pasteurella multocida* and beta-hemolytic Streptococci to be the most common isolates in pure culture but *Escherichia coli* less frequently. Pure cultures are frequently considered to be more likely associated with genital tract infections in the bitch [16], but also in the semen of stud dogs with abnormal clinical and semen findings [41]. However, pure culture does not always result in clinical symptoms [5]. And even further, Bjurström et al. found mostly mixed cultures of two species in a 3-year study on 59 healthy dogs, but they also found pure cultures on at least one occasion in 72.8% of bitches (n = 43) [4]. Nevertheless, *Escherichia coli* isolates found in pure culture were especially associated with vaginitis and puppy death [16], as well as pyometra [42]. *Escherichia coli* and beta-hemolytic Streptococci were identified in high growth intensities more often than other genera in our study. They are described to be correlated with reproductive disease in bitches [16], but also clinical andrological findings and semen alterations [41], as well as teratozoospermia in male dogs [43]. Due to the lack of anamnestic information (healthy/suspected reproductive-diseased) about the bitches sampled, the clinical relevance of higher growth intensities of *Escherichia coli* and beta-hemolytic Streptococci remains questionable. However, other studies found these bacterial species in high-grade growth also on the vaginal mucous membranes of clinically healthy dogs [9,13]. Additionally, beta-hemolytic Streptococci, also *Staphylococcus intermedius* in pure cultures, could be associated with vaginitis in bitches [16]. It is, however, noteworthy that according to Weese and van Duijkeren [44], many canine isolates reported as *Staphylococcus intermedius* might, in fact, be *Staphylococcus pseudintermedius.* The reason for this is that phenotypic tests were used for characterization, but differentiation would require molecular methods. For this reason, today, Staphylococcus intermedius, Staphylococcus pseudintermedius, and *Staphylococcus delphini* are classified as *Staphylococcus intermedius-group*, with individual differentiation often being performed by MALDI-TOF. According to the authors’ experience, differentiation between the above-mentioned species by MALDI-TOF is not reliable in all isolates.

Despite the fact that applied semiquantitative scoring is not directly comparable to quantitative assessments of bacterial growth, it is not uncommon [4,5,6,45], and it still allows for the conclusion of increased bacterial growth in culture. It remains, however, open whether the increased proof of high-grade growth and/or monoculture might be related to storage, shipment, and processing of samples (usually overnight), sampling technique (often without speculum in the field) and site of sampling within the vagina [8], different technicians and laboratory habits (individual variation), antimicrobial pre-treatment (selectively enriching resistant bacteria) [2] or even breed [46]. Previously, samples from the posterior vagina [8], taken with culture medium-moistened swabs [4] and from long-haired breeds [46], had been shown to have more likely enriched bacterial growth. Besides storage, etc., conditions had been confirmed to have an impact on bacterial findings on the microbiome composition in metagenomics analyses [47], as well as conventional culture (Siesenop and Verspohl, unpublished data). This strongly supports the need for further studies.

Despite the overall good agreement of our results with earlier data on healthy bitches, the lack of information about the clinical history of the bitches and about the details of sampling (with/without a speculum, dry/moistened swab) included are a non-negligible limitation of our study, independent of the huge sample size. The lack of information includes the reason for the presentation and the health status as well as the stage of the estrus cycle. Without a doubt, a certain percentage of bitches had been collected for routine breeding soundness examination before mating or artificial insemination, either on bitch or stud owner’s request or on veterinary recommendation [48]. However, vaginal swabs of some bitches might have been collected because of suspected reproductive disease due to clinical signs, e.g., vaginal discharge. Of those, some might have suffered from clinical illness or at least signs of inflammation evaluating vaginal cytology, even though the occurrence of clinically apparent vaginitis is quite uncommon in adult bitches [49]. Some samples might have been taken from the vagina in the case of open cervix pyometra, with *Escherichia coli* being the most common pathogen isolated in this context [42], possibly explaining the high frequency of high-grade growth in monoculture. Otherwise, some bitches might have been sampled for suspicion of illness but might not have had pathological but physiological discharge and no inflammatory signs in vaginoscopy and/or vaginal cytology. Nevertheless, at least some of these bitches are treated in the field without proper diagnosis, including vaginoscopy, vaginal cytology, and/or ultrasound of the genital tract based on findings in bacterial aerobic culture. It has been repeatedly shown, however, that the microflora of diseased and healthy bitches is alike [5,13], stressing the need for thorough and detailed gynecological examination. This is especially important as in the dog, only Brucella canis is considered obligate pathogenic [17,18,28], whereas the majority of bacteria is either facultative pathogenic or apathogenic. So future studies should include clinical history as well as results of bacterial culture to gain deeper insights and possibly verify differences in the vaginal flora of healthy and (suspected) reproductive-diseased animals. Consequently, despite the large scale of the study, our present investigation can solely ascertain the diversity of the canine vaginal bacteria as identified by aerobic culture techniques but does not allow for conclusions on a physiological flora or even a differentiation between physiological and pathological.

## 5. Conclusions

Our data analysis, including 23,254 vaginal swabs from as well healthy as (suspected) reproductive-diseased bitches correspond well to previous results obtained from a limited number of healthy animals. This supports that based on conventional aerobic bacterial culturing, the canine vagina is cohabited by various bacteria, such as *Escherichia coli*, beta-hemolytic Streptococci, coagulase-positive Staphylococci, Pasteurellales, *Streptococcus* spp. (other than beta-hemolytic) and aerobic sporulators. A major limitation of this retrospective study is the lack of background information on the bitches health status and cycle stage, but also on sampling techniques; further prospective studies using culture techniques, but also next generation sequencing should take these aspects, but also subsequent fertility into consideration to better characterize the canine vaginal flora and to identify a possible impact of the vaginal microflora on fertility in healthy bitches. Due to the current inability to differentiate between physiological and pathological vaginal flora, antimicrobial treatment should only be considered in case of concomitant signs of inflammation as identified by gynecological examination, but not solely on the presence of selected bacterial species.

## Figures and Tables

**Table 1:** Grouping of bacterial species isolated from canine vaginal swabs.

Group Name	Species
*Escherichia coli*	*Escherichia coli*
Beta-hemolytic Streptococci	e.g., *Strep. canis, Strep. equi* ssp. *zooepidemicus, Strep. equisimilis, Strep. dysgalactiae*
Coagulase-positive Staphylococci	*Staph. aureus, Staph. intermedius, Staph. pseudintermedius, Staph. delphini*
Pasteurellales	*Pasteurella* spp., *Neisseria* spp., *Haemophilus* spp., *Mannheimia* spp.
Common inhabitants of mucosae	*Bacillus* spp., *Streptococcus* spp. (except beta-hemolytic), *Aerococcus* spp., *Weissella* spp., *Lactobacillus* spp., *Lactococcus* spp., *Carnobacterium* spp.
Enterobacterales	*Citrobacter* spp., *Cronobacter* spp., *Enterobacter* spp., *Erwinia* spp., *Klebsiella* spp., *Kosakonia* spp., *Leclercia* spp., *Lelliottia* spp., *Pantoea* spp., *Pluralibacter* spp., *Providencia* spp., *Proteus* spp., *Rahnella* spp., *Raoultella* spp., *Serratia* spp., *Morganella* spp., *Hafnia* spp., *Escherichia* spp. (except *Escherichia coli*), *Kluyvera* spp., *Salmonella* spp., *Yersinia* spp., *Pseudescherichia* spp., *Buttiauxiella* spp., *Mixta* spp.
Gram-negative non-fermenters	*Pseudomonas* spp., *Burkholderia* spp., *Acinetobacter* spp., *Achromobacter* spp., *Bergeyella* spp., *Psychrobacter* spp., *Stenotrophomonas* spp., *Empedobacter* spp., *Sphingobacterium* spp., *Sphingomonas* spp., *Pseudoacidovorax* spp., *Ralstonia* spp. *Ochrobacterium* spp., *Moraxella* spp., *Flavimonas* spp., *Chryseobacterium* spp., *Elizabethkingae* spp., *Advenella* spp., *Comamonas* spp., *Alcaligenes* spp.
*Enterococcus* spp.	*Enterococcus* spp.
Coagulase-negative Staphylococci and other Micrococcaceae	*Staph.* spp. (except for above listed), *Rothia* spp., *Micrococcus* spp., *Macrococcus* spp., *Arthrobacter* spp., *Glutamibacter* spp.
Aeromonales	*Aeromonas* spp.
Gram-positive rods *	*Actinomyces* spp., *Streptomyces* spp., *Paenarthrobacter* spp., *Rhodococcus* spp., *Trueperella* spp., *Pseudarthrobacter* spp., *Corynebacterium* spp.

* Gram-positive rods, except for *Bacillus* spp., *Lactobacillus* spp., and *Carnobacterium* spp.

**Table 2:** Number of isolates per sample within 23,254 canine vaginal swabs in total. Results are given as the total number of samples (n) as well as the respective percentage containing 1, 2, 3, etc., species per sample.

Number of Bacterial Species Per Sample	n	Percentage
1	10,158	43.7%
2	8302	35.7%
3	2326	10.0%
4	323	1.4%
5	13	0.1%
6	3	0.01%
not specified *	1263	5.4%
No growth	866	3.7%

* mucosal flora, as defined by each laboratory.

**Table 3:** Percentage of monocultures isolated from canine vaginal swabs within each group of bacterial species. Results are given as the total number of monocultures within a group (n), as the percentage of a group within the group of all monocultures (percentage of overall monocultures), as well as the percentage of monocultures for each group (percentage of monocultures within a group) * gram-positive rods, other than *Bacillus* spp., *Lactobacillus* spp. and *Carnobacterium* spp.

Group of Bacterial Species	n	Percentage of Overall Monocultures	Percentage of Monocultures within a Group
*Escherichia coli*	2976	29.3%	38.3%
Pasteurellales	1779	17.5%	35.8%
Coagulase-positive Staphylococci	1695	16.7%	28.4%
Beta-hemolytic Streptococci	1583	15.6%	24.2%
Enterobacterales	674	6.6%	27.2%
Gram-negative non-fermenters	552	5.4%	25. 9%
Common inhabitants of mucosae	519	5.1%	16.0%
*Enterococcus* spp.	191	1.9%	17.7%
Coagulase-negative Staphylococci and other Micrococcaceae	186	1.8%	26.7%
Aeromonales	2	0.02%	9.5%
Other gram-positive rods*	1	0.01%	7.7%

**Table 4:** Comparison of qualitative analysis of bacterial species in each year (2015–2021), results are presented as percentage (%) of samples per group, specimens containing “mucosal flora” (as defined by the laboratory) were excluded.

Group of Bacteria	2015	2016	2017	2018	2019	2020	2021
*Escherichia coli*	21.70	21.36	18.72	22.85	23.40	23.34	23.35
Beta-hemolytic Streptococci	19.07	18.43	18.23	18.87	19.14	19.48	17.78
Coagulase-positive Staphylococci	14.95	16.32	17.03	17.17	17.12	19.22	16.90
Pasteurellales	15.91	14.19	14.93	13.89	13.64	12.82	15.02
Common inhabitants of mucosae	9.90	10.48	13.19	7.65	8.25	7.27	9.57
Enterobacterales	6.86	7.40	6.51	7.91	6.87	6.99	7.07
Gram-negative non-fermenters	6.09	5.64	5.47	6.10	6.51	6.04	6.55
*Enterococcus* spp.	2.71	3.01	3.10	3.47	3.50	3.36	2.33
Coagulase-negative Staphylococci and other Micrococcaceae	2.60	3.13	2.73	2.02	1.44	1.40	1.33
Aeromonales	0.17	0.03	0.09	0.03	0.09	0.06	0.00
Gram-positive rods *	0.03	0.00	0.00	0.02	0.05	0.04	0.10

* gram-positive rods, other than *Bacillus* spp., *Lactobacillus* spp., and *Carnobacterium* spp.

## Data Availability

The data presented in this study are available on request from the corresponding author.
